# Supplementary figures and images for: The pleasurable urge to move to music is unchanged in people with musical anhedonia
Source: PLoS One. 2025 Jan 7;20(1):e0312030. doi: 10.1371/journal.pone.0312030 (PMC11706506; doi:10.1371/journal.pone.0312030)

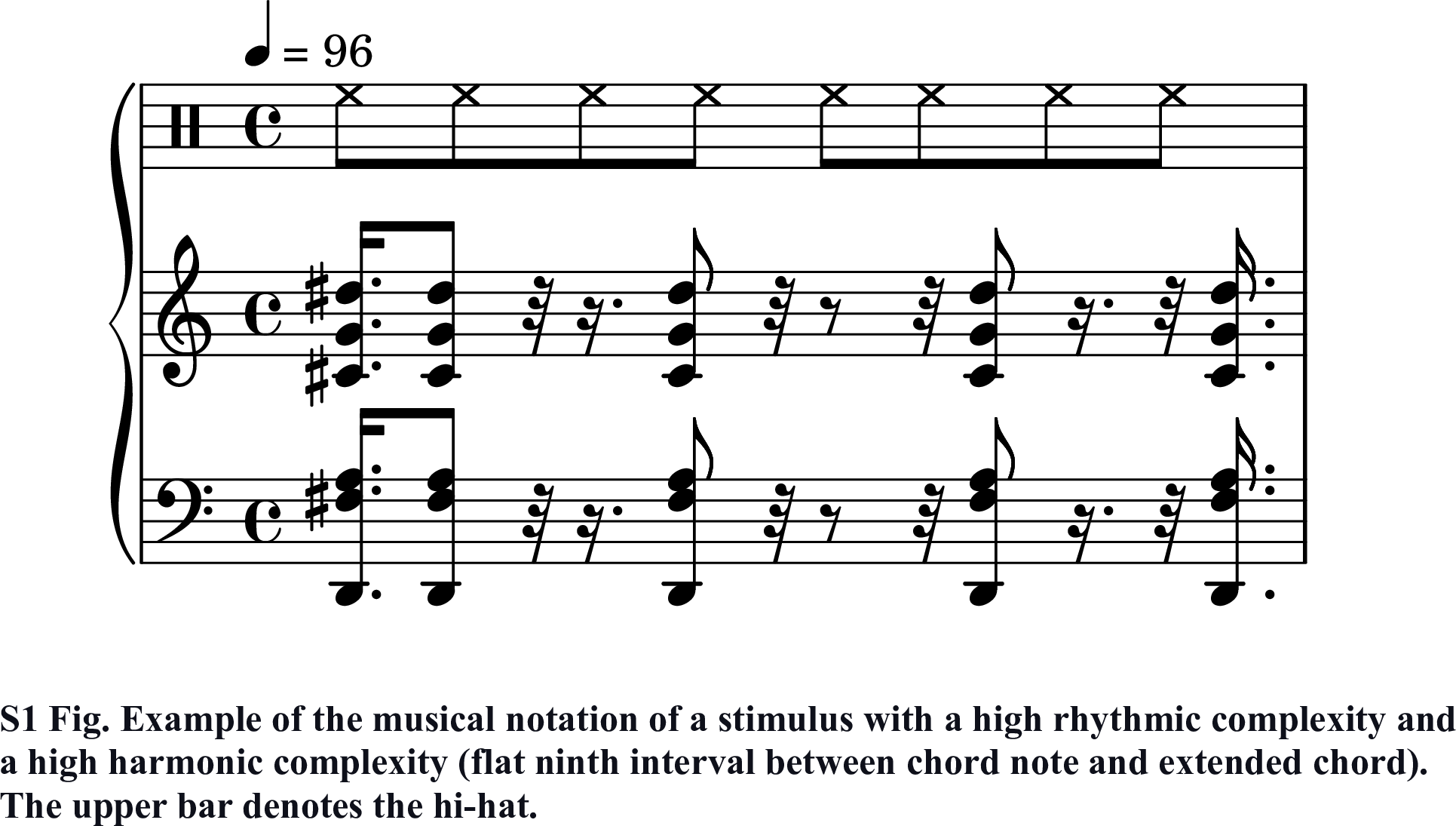

Supplement: S1 Fig — (TIF) [file pone.0312030.s002.tif]

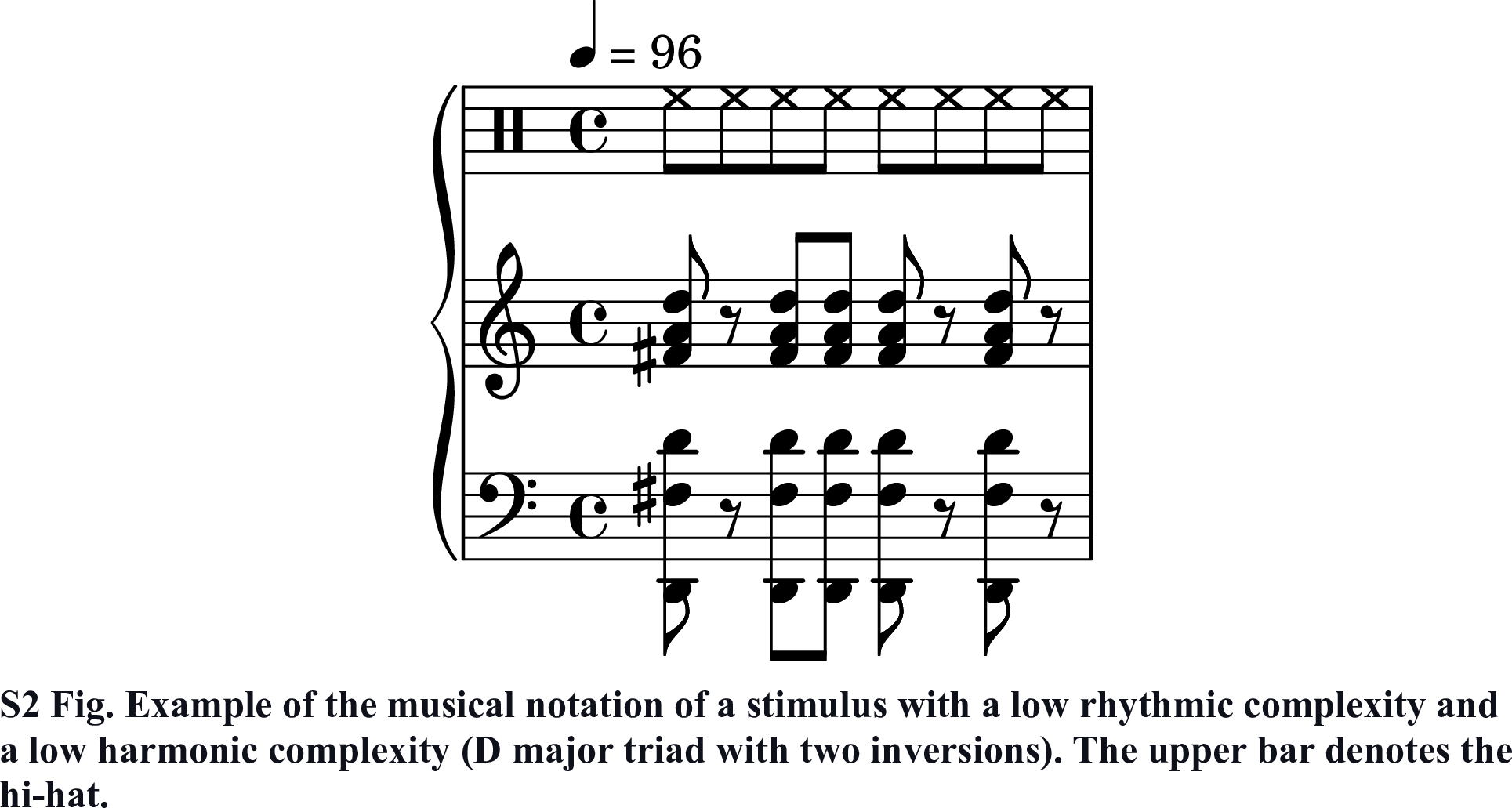

Supplement: S2 Fig — (TIF) [file pone.0312030.s003.tif]

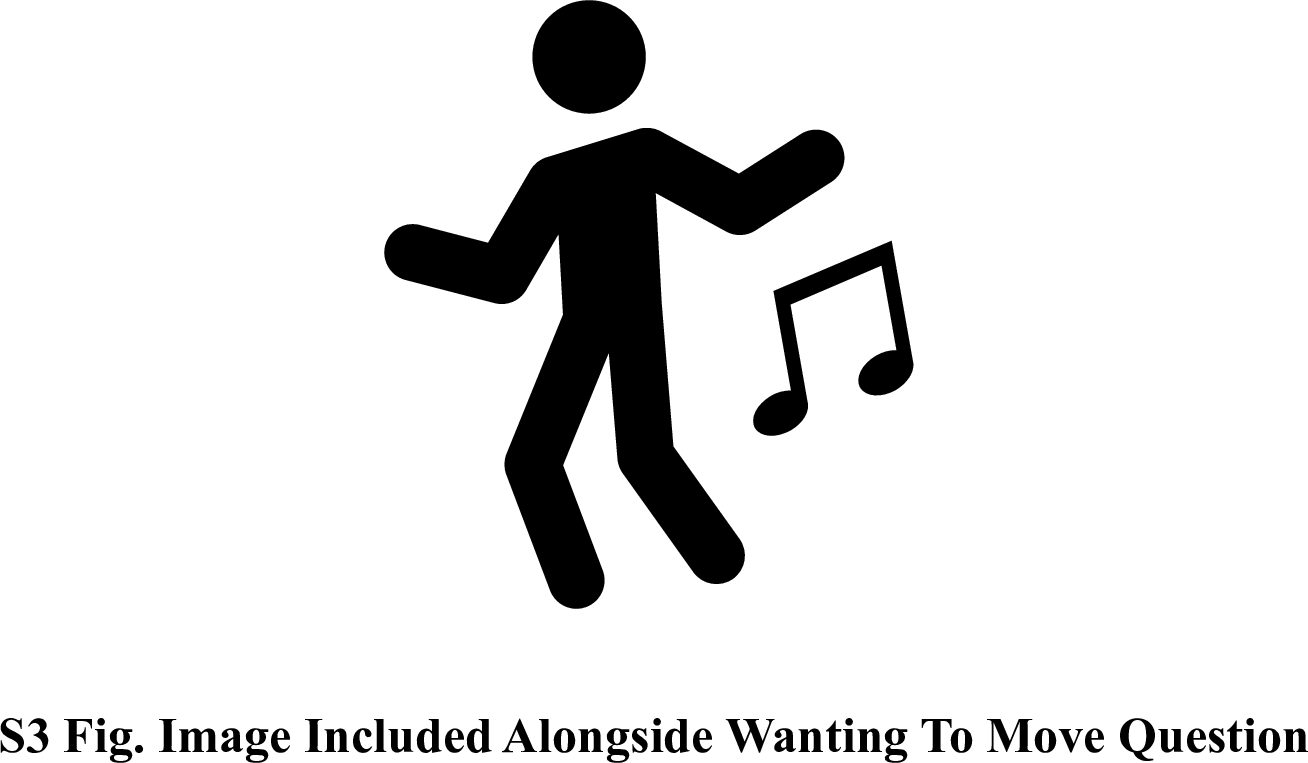

Supplement: S3 Fig — (TIF) [file pone.0312030.s004.tif]

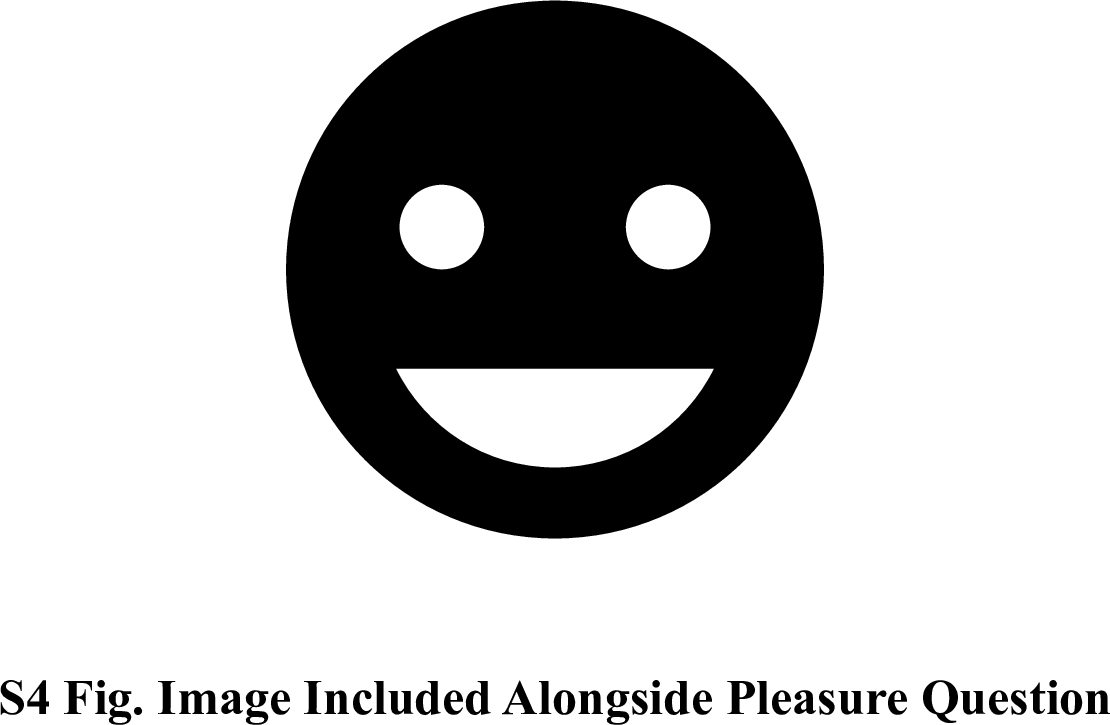

Supplement: S4 Fig — (TIF) [file pone.0312030.s005.tif]
