# Supplementary material for: The pleasurable urge to move to music is unchanged in people with musical anhedonia
Source: PLoS One. 2025 Jan 7;20(1):e0312030. doi: 10.1371/journal.pone.0312030 (PMC11706506; doi:10.1371/journal.pone.0312030)
Supplement: S1 Table — M = mean, SD = standard deviation. (DOCX) [file pone.0312030.s006.docx]

**S1 Table. Demographics**

|  | Age | | Gender | | | Years of Musical Training | |
| --- | --- | --- | --- | --- | --- | --- | --- |
|  | M | SD | Females | Males | Non-Binary | M | SD |
| Musical Anhedonia | 33.12 | 13.87 | 4 | 13 | 0 | 4.29 | 11.03 |
| Matched Control | 33.06 | 11.89 | 4 | 13 | 0 | 4.69 | 10.3 |
| Full Control | 26.05 | 7.76 | 75 | 71 | 2 | 4.42 | 6.3 |

M = mean, SD = standard deviation
