# Supplementary material for: The pleasurable urge to move to music is unchanged in people with musical anhedonia
Source: PLoS One. 2025 Jan 7;20(1):e0312030. doi: 10.1371/journal.pone.0312030 (PMC11706506; doi:10.1371/journal.pone.0312030)
Supplement: S2 Table — Χ2 = Chi-Square, RC = Rhythmic Complexity, HC = Harmonic Complexity, ~ indicates an interaction, Harmonic complexity was compared to the model with rhythmic complexity as a predictor. (PDF) [file pone.0312030.s007.pdf]

Supplementary Table 2.
Likelihood Ratio Test Results

	Pleasure Ratings		*Urge To Move Ratings*	
	χ^2	*p*	χ^2	*p*
Full Control: RC	260.34	< 0.001*	245.63	< 0.001*
Full Control: HC	166.41	< 0.001*	149.09	< 0.001*
Full Control: RC ~ HC	45.58	< 0.001*	14.15	< 0.001*
Musical Anhedonia: RC	19.13	< 0.001*	20.82	< 0.001*
Musical Anhedonia: HC	19.65	< 0.001*	17.22	< 0.001*
Musical Anhedonia: RC ~ HC	2.16	0.142	2.97	0.085
Matched Controls: RC	27.24	< 0.001*	21.21	< 0.001*
Matched Controls: HC	17.87	< 0.001*	9.61	0.002*
Matched Controls: RC ~ HC	1.09	0.296	0.56	0.455

Note. X^2 *= Chi-Square, RC = Rhythmic Complexity, HC = Harmonic Complexity, ~ indicates an interaction, , Harmonic complexity was compared to the model with rhythmic complexity as a predictor*
