# Supplementary material for: The pleasurable urge to move to music is unchanged in people with musical anhedonia
Source: PLoS One. 2025 Jan 7;20(1):e0312030. doi: 10.1371/journal.pone.0312030 (PMC11706506; doi:10.1371/journal.pone.0312030)
Supplement: Attachment — Submitted filename: Response to Reviewers.pdf [file pone.0312030.s008.pdf]

Journal Requirement Questions

1. Please ensure that your manuscript meets PLOS ONE's style requirements, including those for file naming. The PLOS ONE style templates can be found at

   https://journals.plos.org/plosone/s/file?id=wjVg/PLOSOne_formatting_sample_main_body.pdf
   https://journals.plos.org/plosone/s/file?id=ba62/PLOSOne_formatting_sample_title_authors_affiliations.pdf

   The article has been updated to meet PLOS ONE's style requirements.

2. We note that the grant information you provided in the 'Funding Information' and 'Financial Disclosure' sections do not match.

   When you resubmit, please ensure that you provide the correct grant numbers for the awards you received for your study in the 'Funding Information' section

   We have rectified this.

3. Thank you for stating the following financial disclosure: [This research was supported by a grant to VP from the Natural Sciences and Engineering Research Council of Canada (NSERC 2021-04026). IR was funded by the Canadian Graduate Scholarship - Masters by NSERC and by the Fonds de Rechereche du Québec - Nature et Technologies (FRQ - NT). SDB and NF received funding from a Discovery Grant (RGPIN-2019-05453) from NSERC, and by the Canada Research Chair program (CRC in music auditory-motor skill learning and new technologies).]. Please state what role the funders took in the study. If the funders had no role, please state: ""The funders had no role in study design, data collection and analysis, decision to publish, or preparation of the manuscript."" If this statement is not correct you must amend it as needed. Please include this amended Role of Funder statement in your cover letter; we will change the online submission form on your behalf.

   The funders had no role in study design, data collection and analysis, decision to publish, or preparation of the manuscript.

4. Thank you for stating the following in the Competing Interests section:[SDB is on the board of the BeatHealth company dedicated to the design and commercialization of technological tools for assessing rhythm capacities such as BAASTA and implementing rhythm-based interventions.]. Please confirm that this does not alter your adherence to all PLOS ONE policies on sharing data and materials, by including the following statement: "This does not alter our adherence to PLOS ONE policies on sharing data and materials." (as detailed online in our guide for authors http://journals.plos.org/plosone/s/competing-interests). If there are restrictions on sharing of data and/or materials, please state these. Please note that we cannot proceed with consideration of your article until this

information has been declared. Please include your updated Competing Interests statement in your cover letter; we will change the online submission form on your behalf.

Please find the updated statement below.

"SDB is on the board of the BeatHealth company dedicated to the design and commercialization of technological tools for assessing rhythm capacities such as BAASTA and implementing rhythm-based interventions. This does not alter our adherence to PLOS ONE policies on sharing data and materials."

Reviewers' comments:

Reviewer #1: I have mixed feelings about this article. On one hand, it follows a clever idea to separate urge to move and pleasure in groove, and employs a high qualitative research design to do so. On the other hand, the literature review, theoretical underpinning, and discussion are somewhat lacking and there are some issues with the analysis.

We wanted to thank the reviewer for their thorough and constructive comments. We have attempted to incorporate their feedback throughout the manuscript where possible.

Major issues:

1. I see some issues with how the model and variable selection were performed; it would be worthwhile to do some more testing and update the models if necessary.

a) Variable selection: you conducted forward addition, but as far as I know, backwards elimination or bidirectional addition/elimination is more common. The latter is in any case the more thorough method and should be preferred.

b) I don't see a test for the model structure. You state that your baseline model had a by-participant intercept, which is reasonable for a start - although you do not provide your theoretical reasoning for it. However, it seems you did not check for other options. It is counterintuitive to expect that the stimuli are sufficiently described by HC and RC, and that a by-stimulus intercept would thus be unnecessary. Similarly, a slope for RC can be expected: why would every participant react with the same strength to the manipulations of RC? I did a quick test for some of the possible configurations with your data, and it seems that a model with (Rhythm | ID) + (1 | MusicName) would be the most efficient model structure (but I might have made a mistake, and I did not check for the groups independently!). I guess this model will run into convergence issues with your MA subset due to the low amount of data. I am also aware that the mediation package is not able to work correctly with slopes. I have not calculated all your models, and can't say whether different models would change some of your results. Yet, I think it is necessary to check for additional model structures, at least for the by-stimulus intercept. If you choose to ignore better fits with slopes, you can probably find an argumentation for that, as a) it is not uncommon to skip the respective checks, i.e., provide an argumentation that other

papers did not use it and why you think this is the correct way to handle it or b) mediation analysis is central to your study and, as far as I know, this gets a bit messy with random slopes, i.e., provide an argumentation based on your intended method and why you think results are not changed by dropping down to your current model.

We wanted to address these points together as similar reasoning was used for both. While bidirectional addition/elimination is generally the most preferred method in exploratory analyses, it is less preferred for hypothesis-driven analyses (Harrell, 2001). Additionally, we chose a forward-addition model instead of a backward-elimination model because of model convergence issues. Because we wanted to replicate the findings of Matthews et al., 2019, we wanted to include harmonic complexity in the model. As you mentioned in your comment, the maximal model is (Rhythm | ID) + (1 | MusicName). Unfortunately, the model, which includes harmonic complexity as a fixed effect, fails to converge. Therefore, to address our hypothesis, we felt it was better to build the model up in a forward addition process to ensure convergence.

For the mediation analyses, as you mentioned in your comment, unfortunately, random slopes do not work properly in the mediation package. Although it could be ideal to incorporate random slopes, we believe that the mediation analysis is a central finding of the paper and thus have included it over the random slopes.

References:

Harrell, F. E. (2001). *Regression modeling strategies: with applications to linear models, logistic regression, and survival analysis* (Vol. 608). New York: springer.

Matthews, T. E., Witek, M. A., Heggli, O. A., Penhune, V. B., & Vuust, P. (2019). The sensation of groove is affected by the interaction of rhythmic and harmonic complexity. *PLoS One*, *14*(1), e0204539.

2. The paper is at times very repetitive. In some cases, you are repeating half sentences that you just brought up one or two sentences above. In others, full sentences are almost duplicates. It would enhance the readability of your paper if you could cut down on repetitions.

We have reviewed the manuscript and attempted to eliminate repetitions.

3. Your literature review is one-sided (even more emphasized by frequent mentions of the own lab) and misses some studies that examined the relationship of pleasure and urge to move that should be taken into account. For example, Senn et al. (2020) tried to separate urge to move and pleasure through a specific set of real music stimuli but failed, probably due to methodological problems. In Senn et al. (2023), the correlation between pleasure and urge to move was rather low and pleasure hardly predicted any variance in urge to move ratings, again using stimuli selected to show independence. And probably of most interest to your study is the psychological model of groove (Senn et al., 2019; later adapted) that postulates a so far untested for hedonistic feedback loop between urge to move and pleasure in groove. Their reasoning and argumentation

could be quite useful for your introduction and discussion, especially as your study seems to confirm this loop/the half that has not been tested before. In my opinion, your study would benefit greatly from considering these results – and not including some of these leaves the impression that of a hastily literature review process.

Thank you for bringing these articles to our attention. We have now incorporated them into the Introduction. In particular, Senn's 2019 model of groove fits well alongside our findings, and as such, we have also included it in the Discussion section.

a) You are not sufficiently introducing the idea of a non-separable urge to move and pleasure. On what basis would you think that they would be either the same thing or fuse to a single entity? In case it is because of the high correlation, then an r = 0.8 means that pleasure still cannot explain 36% of the variance in urge to move (and this amount likely increases if you add a random intercept). Hence, I don't think this is sufficient as an explanation. And besides this strong correlation, Senn et al. (2020) and Düvel et al. (2021) found that the best fitting CFA on their 6-item questionnaire on groove has in fact two constructs (pleasure and urge to move), and not one. This is quite a good argument that they are separable already.

We have now expanded our discussion including, Senn, 2020 in this section and expanded the discussion of the possible separability of pleasure and the urge to move in the sensation of groove.

b) Pleasure is not sufficiently introduced in the introduction nor discussed later. You provide some hints that you subscribe to the idea that multiple pleasures exist or at least pleasure derived from multiple sources. Yet, as this is quite important for your main result and conclusion, this is underdeveloped and lacks proper sources about the nature of pleasure. I'm not aware of a published study that investigates the nature of pleasure in groove in particular; there are some things in Danielsen (2006), Roholt (2014), Janata et al. (2012) and Bechtold et al. (2023) but the last two seemingly preferred the term "positive affect" and I'm not sure if it fits completely with your concept of pleasure. However, there are countless texts on pleasure from many disciplines and your paper would benefit from being clearer on how you actually understand one of the examined core concepts.

We agree with the reviewer that pleasure is a complex topic, and that it likely originates from various sources. In the section on predictive coding (i.e., L81-98), we have expanded on the idea that pleasure from groove originates from the processes of learning and refining of internal models. Although there are other ways to define and understand pleasure, this is the conceptualization that motivated the current study.

c) Musical Anhedonia is also not properly introduced. As a reader, I'm sufficiently informed how you operationalized it in the study, but what it actually is, how it comes about, what body or brain parts it affects, what consequences it has for affected people, etc. is not discussed at all.

Music specific anhedonia has been observed in the general population (congenital anhedonia) and in people with brain injury (acquired anhedonia). Currently, there is no information about genetic contributions to this trait. The very few neuropsychological studies of acquired

anhedonia have found no consistently overlapping brain regions, with auditory, parietal, insula and amygdala known to be damaged in individual cases (Belfi & Loui, 2020). A brain imaging study of congenital amusia showed reduced activity in auditory and reward regions, along with decreased white matter connectivity between the primary auditory cortex and reward networks (Martínez-Molina et al., 2016). This additional information has been added to the Introduction on lines 130-133.

References:

Belfi, A. M., & Loui, P. (2020). Musical anhedonia and rewards of music listening: Current advances and a proposed model. *Annals of the New York Academy of Sciences*, *1464*(1), 99-114.

Martínez-Molina, N., Mas-Herrero, E., Rodríguez-Fornells, A., Zatorre, R. J., & Marco-Pallarés, J. (2016). Neural correlates of specific musical anhedonia. *Proceedings of the National Academy of Sciences*, *113*(46), E7337-E7345.

4. The method section is very long and, in my opinion, not properly organized. You start with a pseudo-procedure, then go to participants which features many things that have not been introduced before. Then you have a section about rating groove that mostly discusses the stimuli. Afterwards, there is a very long section about measures, followed by a very short section about measures. Then, you have a proper procedure and a data analysis section. My suggestion to make this more streamlined: drop the initial procedure, and start with a section about all measures, then stimuli, then a proper procedure and last the analysis. And regarding the measures, I think you are giving way too many details that are not particularly interesting to the reader. Their main purpose seems to be to check whether a participant has MA and are consequently not discussed much in the results or discussions. I would suggest to significantly cut down on this (e.g., no need to report the exact procedure, stimuli, nor analysis for these if they are established tests) and put the rest in a supplementary file.

We agree that the Methods section could have been better organized. Based on the reviewer's suggestions, we have revised and shortened it.

Minor issues:

Title:
I'm not a native speaker, but to me the title sounds incomplete. Unchanged from what? And is "in Musical Anhedonia" correct? Shouldn't it be "in People with Musical Anhedonia" or something similar.

We have revised the title to: **The Pleasurable Urge to Move to Music is Unchanged in People with Musical Anhedonia**

Abstract:
The abstract is too long and detailed. It should serve as a quick overview over the study and would benefit from being shorter and the main takeaways presented earlier.

The Abstract conforms to the length guidelines of the journal. As part of our revisions, we have made some changes to improve readability.

o L38: I do not think that N=148 qualifies as large and you do not lose anything by cutting this claim. Especially, N = 17 is not large.

The word “large” was omitted from the abstract.

o L38/L44: you are describing your stimuli as rhythmic but then later they apparently have harmonic complexity. It is clear in the paper but incomprehensible in the abstract.

We have altered how we describe our stimuli in order to be more explicit that they vary in both rhythmic and harmonic complexity.

o L47: “response” is very technical, how about “sensation” or “experience”?

“Response” has been changed to “sensation”.

Introduction:
- L51: “often generates a…” is too vague.

We have altered the sentence to say “…of listening to music is that it is often accompanied by a pleasurable urge to move along.”

- L64: please provide the citations after each structural component.

Relevant citations have been included after each structural component of groove is mentioned.

- L71: predictive coding and groove: this theory is en vogue and it might have some importance to your study and thus warrant inclusion. However, the way you summed it up, it does not provide a useful explanation for how groove comes about. If I take just your information, it sounds like I cannot ever experience groove with music where my prediction error is very low (e.g., because I know it very well). This is counterintuitive (and against results regarding familiarity) and the explanation should be corrected to avoid such misunderstandings.

We have expanded on our descriptions of predictive coding by discussing how the theory explains the inverted u-shaped relationship between syncopation and groove ratings, focusing on the interaction between predictability and surprise. Additionally, we included more information about how predictive coding conceptualizes how the urge to move and pleasure come about in response to this interaction.

- L78ff: the studies you list do not show a great variety of stimuli, and I don't think any qualify as natural excerpts, as they are all synthesized. Also, they are all either drum beats or single rhythm stimuli (as there is no syncopation index for polyphonic music other than drums as far as I am aware). Some studies, e.g., Senn et al. (2018) found a different effect of syncopation, Senn et al. (preprint) found none. And while preprints are to be taken with a grain of salt, the latter includes a reanalysis of the Witek et al. (2014) data that showed that the inverted U is not to be found in the more ecologically valid stimuli, but only when experimenter composed ones are included. Hence, I think your claim about variety and naturalism is problematic.

Thank you for bringing this point up. To rectify this, we have included two citations of two preprints demonstrating the inverted U-shaped curve for naturalistic stimuli (Speich et al., 2022; Speuch et al., 2024). We also included a description of the findings of Senn et al., 2018 & 2024 to ensure that all current evidence is presented to the reader.

References:

Spiech, C., Hope, M., Câmara, G. S., Sioros, G., Endestad, T., Laeng, B., & Danielsen, A. (2022, July 26). Sensorimotor Synchronization Increases Groove. https://doi.org/10.31234/osf.io/fw7mh

Spiech, C., Câmara, G. S., Fuhrer, J., & Penhune, V. (2023, September 14). 4/4 and More: Groove in Uncommon Meters. https://doi.org/10.31234/osf.io/vm4x5

- L83ff: this section could do with better transitions. It starts with an almost exact repetition and a re-introduction of the topic, but then goes on to present two studies in more detail. Yet, why these two studies are of particular importance is not clear until the reader has read the full paragraph on them. This makes for strange changes of subject on a first read. It would be beneficial to mark the purpose of the section (no reintroduction needed) and why each study is important for the present study's hypotheses and methods.

We have reorganized this paragraph to avoid repetition and improve the transitions.

- L110: you keep talking about the stimuli, but the reader does not know anything about them yet, besides that they have chords that mark the rhythm. If the stimuli are of such importance here already, please introduce them properly.

As the stimuli are not of great importance at this point in the paper, we have decided to omit mention of the stimuli from this part of the paper.

- L111: here would be a good place to expand on MA

As addressed in point 3c above we have provided additional information about MA on lines 130-133

- L115: here would be a good place to introduce your theory on pleasures/pleasure derived from multiple sources.

As addressed in point 3b above, we added further explanation about how pleasure arises from groove on lines 81 to 98.

Methods:

- L133: "musical history" is misleading, "musical background" instead?

We have changed "musical history" to "musical background".

- L161: misleading headline, this paragraph (until L183) should be titled "stimuli."

The headline of this paragraph was altered to "Stimuli" in order to avoid confusion.

- L188: it is unclear whether the two questions were asked on the same page, on following pages, or in separate blocks of the experiment (which is potentially important for the correlation).

We have clarified in the text that the questions were asked sequentially and on separate pages.

- L188f: what images? Can these be included in the supplementary files?

Please find the two images included as supplemental figures as S3 and S4 Fig.

- L205: a duration of 20 "quarter notes": "beats" might be more appropriate?

"Quarter notes" was changed to "beats"

- L215: here is info about duration, but you don't give this for all tasks (redundant if you decide to cut).

This has been removed and is no longer relevant.

- L304ff: please cite the packages that you use properly.

All packages used have now been properly cited.

- L305: do you mean “Satterthwaite”?

This typo has been addressed

- L312: “that analysis”: it is unclear what is meant by that.

Corrected.

- L311: it seems that you added RC and HC always as quadratic. Please state so if this is true.

A sentence was added stating that RC and HC were always included in the model as a quadratic

Results:
- L333: RC > HC: please provide statistical proof.

We have omitted the line stating that the quadratic effect of rhythmic complexity was stronger than harmonic complexity as this statement was made on the basis of qualitative observations.

- L374: DE remained significant, but also small/tiny. You have not defined boundaries for interpretation in your text, but maybe it would be good idea to add whether you think it is a relevant effect or neglectable.

We revised this on lines 361 and 363.

- L381: here, you have much, much smaller chi2 compared to the controls. Is this subjectable to the small amount of data or would you have another interpretation (e.g., smaller effect)? In general, I think N = 17 is rather small for a model with random by-participant intercept. Did you perform a power analysis beforehand to check whether your theorized for effect size can be found with this number of participants?

It is possible that the smaller chi2 in the anhedonic sample compared to the full control sample is due to the small sample size. Thus, we believe it would be difficult to interpret differences in effect size with such large differences in sample size.

Estimating sample size based on previous work is difficult in this case. The only other behavioural study investigating MA out at the time we designed the study has 10 participants (Mas-Herrero et al., 2014). Further, this study used reward judgements in music buying vs gambling tasks, which are not easily comparable to our study using stimulus ratings. Collecting large samples of people with MA is difficult, but we hope to replicate our findings in a new study this year.

References:

Mas-Herrero, E., Marco-Pallares, J., Lorenzo-Seva, U., Zatorre, R. J., & Rodriguez-Fornells, A. (2012). Individual differences in music reward experiences. *Music Perception: An Interdisciplinary Journal*, *31*(2), 118-138.

Mas-Herrero, E., Zatorre, R. J., Rodriguez-Fornells, A., & Marco-Pallarés, J. (2014). Dissociation between musical and monetary reward responses in specific musical anhedonia. *Current biology*, *24*(6), 699-704.

- L398: please test whether the correlations are significantly different and include the results as proof.

Analyses showing that the correlations are significantly different are reported on lines 414 to 416.

- L405: what is metric complexity in this study?

"Metrical complexity" has been changed to "rhythmic complexity"

- Table 2-4: please make it clearer where one model ends and the other starts, e.g., with a vertical line.

Additional lines have been added to Tables 2-4 in order to make them easier to read.

- L415: you are jumping between presenting results for MA and comparing to the controls. You gave a lot of space to present controls beforehand. I suggest doing the same for the MA group and then turn to the comparison. That way, it is easier for the reader to know what the current topic is.

We reorganized the Results section to discuss the group analyses and then comparisons.

- L433: You hypothesized this? I may have missed it in the introduction, but it seems that it wasn't clear enough then. Or is this a new hypothesis that you bring up here to explain why your

primary hypothesis was not confirmed? If so, it belongs into the introduction, backed by a reasoning.

We have revised the Introduction to better describe this hypothesis and the rationale behind it

- L444: checking with the matched control group is not a clean test. A power analysis relies on an assumed effect size. It is conceivable that the effect is smaller in MA than in the control and thus cannot be found. I don't think that is the case in your study, but please don't present the comparison as if it would be a reliable statistical test.

We have re-worded this section.

Discussion:
- L477: neither used real music clips.

Thank you for pointing out this mistake! We have added the two citations referenced in the introduction here as well in order to rectify it.

- L477ff: I'm missing an interpretation of the results. There is only the single sentence in L485ff, but there is for sure more to be said.

Throughout the Discussion section, we have included additional interpretations of the findings in regard to the literature as a whole.

- L491: what are groove stimuli?

To reduce confusion, "groove stimuli" has been changed to "musical stimuli that illicit groove"

- L495: groove is maintained through urge to move: this is circular. You have not provided an understanding of groove that exceeds the pleasurable urge to move. Hence, for someone with MA, we would, in theory, assume that either groove equals the urge to move alone, or they cannot experience groove at all. I understand that your results change this theory, but this needs to be unpacked.

We have adjusted the wording of this sentence to be clearer that we are referring to the preserved quadratic relationship for pleasure and urge to move.

- L507: the perception of perception is also circular.

Due to other edits in the manuscript, this sentence has been omitted.

Conclusion:
- L531: both studies emphasize the urge to move in their definitions of groove, not actual movement. However, there are studies that showed that entrained body movement increases pleasure, e.g., Bernardi et al. (2017).

Thank you for bringing this up, as we agree that the distinction between movement and urge to move should be made clear. As such, we have changed the word "movement" to "urge to move".

Reviewer #2:

We wanted to thank reviewer #2 for their comments which have brought up interesting implications and that we feel have allowed us to make the paper stronger.

Your study investigates how anhedonics respond to groove. Your findings--that the pleasurable urge to move in your anhedonic cohort was similar to that of the control group--is revealing and thought-provoking. I have a few methodological concerns: the sound samples are short; and the feeling of urge to move and pleasure is self-reported. I think you need to offer more justification for why this is an appropriate protocol. For instance, is the urge to move instantaneous--or can it sometimes arise after a longer context has been established? Wouldn't the urge to move be more accurately assessed by directly observing the participants? In addition, you only offer one musical example, showing a repeated, syncopated dominant 7th chord. Absent a richer musical context, I'm skeptical about pleasure ratings for harmony based on a single chord--this seems simplistic to me.

While the sound samples we used in our study may be short and responses are based on self-report measures, these same stimuli have been successfully used in other studies investigating the sensation of groove, even showing differences in ratings between musicians and non-musicians (Matthews et al., 2019; Stupacher et al., 2022). Additionally, a brain imaging study used these stimuli to show activity in reward-related regions in response to the medium complexity/high groove stimuli (Matthews et al., 2020).

It is important to clarify that the urge to move can be experienced without physical movement, although moving to the beat has been shown to enhance the sensation of groove (Spiech et al., 2022). It could be interesting to measure physical movement in response to groove in anhedonia, but this is both difficult and muddied by individual differences in people's willingness to move to music in an experimental setting. This could be an interesting direction for a future study.

<u>References:</u>

Stupacher, J., Wrede, M., & Vuust, P. (2022). A brief and efficient stimulus set to create the inverted U-shaped relationship between rhythmic complexity and the sensation of groove. *Plos one*, *17*(5), e0266902.

Matthews, T. E., Witek, M. A., Heggli, O. A., Penhune, V. B., & Vuust, P. (2019). The sensation of groove is affected by the interaction of rhythmic and harmonic complexity. *PLoS One*, *14*(1), e0204539.

Matthews, T. E., Witek, M. A., Lund, T., Vuust, P., & Penhune, V. B. (2020). The sensation of groove engages motor and reward networks. *NeuroImage*, *214*, 116768.

Spiech, C., Hope, M., Câmara, G. S., Sioros, G., Endestad, T., Laeng, B., & Danielsen, A. (2022, July 26). Sensorimotor Synchronization Increases Groove. https://doi.org/10.31234/osf.io/fw7mh

Also, the example is notated poorly rhythmically: in a syncopated rhythm, you should always show where the beat lands: 8th, 16th rest, 16th tied a 16th (to show the beat), 16th rest, etc. I think your Appendix should include more musical examples, since they are a cornerstone of the study.

We acknowledge that these stimuli could be notated differently, but these sample notations have been used in previous work (Matthews et al., 2019), and to avoid confusion across papers, we wish to keep the figures the same. We have added additional notation examples to our supplementary materials (See S1 & 2 Figs).

As a musician, I also found it very difficult to follow how you reported the results: the information is no doubt all there, but it is a puzzle trying to sort through it. For instance, given what you describe, you would expect anhedonics to be indifferent to changes in harmony--since you assert that harmonic complexity influences the urge to move indirectly through its relationship with pleasure, to which anhedonics are presumably indifferent. This seems like an obvious question to me, but I had to read your results several times before I found the answer. I would do more to highlight this finding.

While our previous work indicates that in those with typical music reward responses, harmonic complexity has a stronger effect on pleasure, this appears not to be the case for those with musical anhedonia. Therefore, we hypothesize that those with musical anhedonia are most likely using harmonic complexity differently from their typical hedonic peers. Future work should investigate this idea further.

Also, in line 197, you mention that the BMRQ evaluates sensory-motor reward responses. Given your findings, do anhedonics report higher ratings there than for other sub-scales? If not, how do you explain that?

To investigate this point further, we compared the BMRQ subscale scores across the musical anhedonia group and found that the Sensory-Motor score was not higher than the others. This may be because this subscale has only three questions, two of which are related to dance, which also contains a social component. Thus, it may be tapping into other aspects beyond just sensory motor enjoyment of music.

I have a similar question about your statement in line 472. The majority of popular music is groove oriented. If "the desire to move may itself generate pleasure," wouldn't anhedonics generally enjoy popular music? If they don't, how do you square that with your findings?

This is an interesting question that we cannot answer with our current findings alone. Evidence from two types of future studies could help us to answer this question. First, we can test whether individuals with musical anhedonia respond differently to real musical stimuli than experimenter-constructed stimuli. There is more musical information in real music clips, and this additional information may alter the groove response of those with musical anhedonia. Second, we could investigate the genre preferences of those with musical anhedonia. In a currently planned follow-up study of this experiment, we will use naturalistic stimuli and collect information on genre preference to address these questions.

Overall, I think you should establish more convincingly how your experimental protocol reflects real world musical experiences and, given the inter-disciplinary topic, more clearly explain your results.

A few small comments:
Line 91 - this should read "altering the content of the chords" (tonality refers to the key, which isn't what you mean).

We have changed the sentence as per your suggestion.

Line 177- I don't know what you mean by "an extension"--in music, that refers to lengthening a phrase, which isn't the case here.

Thank you for catching this, we meant to state that it was an extended chord. We have changed this throughout the manuscript.

Line 248: Using the word "difference" and "differ" in the same sentence is confusing. I would rewrite this as "The tests differ in the way the oddball examples are formed" or something like that.

Due to a comment made by Reviewer #1, we have omitted this sentence.

Reviewer #3: sharpen the data analysis and discussion of this research, pay attention to the level of relevance of the method, problems can be sharpened again so that the findings and impact of this research are very large

By addressing the comments from reviewers #1 and 2, we believe we have most likely addressed the non-specific comments of Reviewer #3.
