# Supplementary material for: The pleasurable urge to move to music is unchanged in people with musical anhedonia
Source: PLoS One. 2025 Jan 7;20(1):e0312030. doi: 10.1371/journal.pone.0312030 (PMC11706506; doi:10.1371/journal.pone.0312030)
Supplement: Attachment — Submitted filename: Response To Reviewers #2.pdf [file pone.0312030.s009.pdf]

Reviewer #1: I would like to thank the authors for their careful revision. All my points were addressed (except for one thing, see below) and I think the manuscript is in a much better state in its current form.

The one issue I still have is transparency with modelling decision. I can fully comprehend that you selected the variables based on previous studies, and that you dismissed slopes and an additional intercept as you wanted to stick with the mediation package. Yet, I think it needs to be stated in the paper as well, not just hidden in the answer to a reviewer. Similarly to what I said in my original comment: you can add a statement that you preselected the variables based on theory/desire to replicate previous findings and did not check for other options or combinations. And you can make the argument that the package (and low amount of MA participant) prohibits using slopes/MusicName intercept. But, as I said, this needs to be made clear to the reader, not just the reviewer.

I trust the authors and editor that this comment is incorporated or dismissed without the need for another round of reviews.

On a personal note, I think that convergence issues shouldn't play a role in study with sufficient funds in the background and universities that have staff that can provide help with statistics. They can usually be avoided through better modelling, changing the statistical framework, stronger experimental design, or having more participants. Simulating the statistical power beforehand can give you an indication whether such changes to your plans are necessary. But sometimes, the actual data differs from expectations and the analysis must adapt accordingly. It is too late for this study, but for future work, it might be beneficial to consult with a statistician what options you have with your data specifically. In my eyes, it's has been a malpractice for the past years in music psychology to not test more rigorously for model structure. I can only suspect that convenience (i.e. avoiding such convergence issues) or a learned disregard due to the often small changes in results (until you find yourself with data in which slopes are crucial for a correct picture) are the motivations behind this. As a minor note: (Rhythm | ID) + (1 | MusicName) is not at all the maximal structure, it is what I found to be the most efficient one in a quick test. The maximum would be along the lines of (Rhythm * Harmony | ID) + (Rhythm * Harmony | MusicName).

We want to thank reviewer #1 for their comments. We have added sentences (L310 - 314) to be completely transparent regarding our decisions about our models. Additionally, we wanted to thank the reviewer for their personal note, and we will consider this advice for future studies.

Reviewer #2: Thank you for addressing many of questions raised in the initial review. I still have concerns about the methodology, and feel you should include some evidence that

urge to move is evident within 10 seconds and remains constant when the duration is longer--i.e. situations in which the urge to move would only arise after 10 secs for an unchanging stimulation are rare/unlikely.

Previous studies have utilized samples of a variety of lengths, both longer and shorter than 10 seconds, and elicited groove (Leow et al., 2014; Spiech et al., 2022 [Preprint]; Witek et al., 2014. The other component of your question, if the urge to move remains constant over an extended period of time, has yet to be tested. This would be an interesting question to investigate in future studies but is outside the scope of the present study.

References:

Leow, L. A., Parrott, T., & Grahn, J. A. (2014). Individual differences in beat perception affect gait responses to low-and high-groove music. *Frontiers in human neuroscience*, *8*, 811.

Spiech, C., Hope, M., Câmara, G. S., Sioros, G., Endestad, T., Laeng, B., & Danielsen, A. (2022, July 26). Sensorimotor Synchronization Increases Groove. https://doi.org/10.31234/osf.io/fw7mh

Witek, M. A., Clarke, E. F., Wallentin, M., Kringelbach, M. L., & Vuust, P. (2014). Syncopation, body-movement and pleasure in groove music. *PloS one*, *9*(4), e94446.

I also think it would be worth justifying using subjective measures to measure the urge to move, as opposed to direct observation. For instance, have previous studies shown a high correlation between the two?

As stated in our previous response to this question, for this study, we are focusing specifically on the urge to move, not movement itself. While previous work has found that the two are related (Janata et al., 2012; Spiech et al., 2022), an individual may experience an urge to move to the music without physically moving to the music at all. By its very nature, the urge to move is a subjective experience, and as such, it must be assessed via subjective measures. Future work could investigate the role movement may play in this relationship, but this part is outside the scope of the current study.

References:

Janata, P., Tomic, S. T., & Haberman, J. M. (2012). Sensorimotor coupling in music and the psychology of the groove. *Journal of experimental psychology: General*, *141*(1), 54.

Spiech, C., Hope, M., Câmara, G. S., Sioros, G., Endestad, T., Laeng, B., & Danielsen, A. (2022, July 26). Sensorimotor Synchronization Increases Groove. https://doi.org/10.31234/osf.io/fw7mh

There are several typos:
line 70: wanting to move

This has been corrected.

86: musical structure

This has been addressed.

92: Meanwhile, in complex rhythms (I don't think you can start a sentence with "While")

We have altered the beginning of the sentence to improve readability.

93: the inverse is true: ... (colon, not comma)

The comma has been replaced by a colon.

199: check grammar here

This has been addressed.

258: awkward wording - I would say "Lastly, high-complexity added a flat ninth above the root of the chord."

We have altered the sentence per your suggestion.

262: FIGURE 1 is not a four-note chord: it is a ninth chord D-F#-A-C-E. This is a rather concerning mistake. Is it misnotated? Or is this the actual chord that was played for participants?

We think this is an issue of terminology. This the actual chord that was played to the participants. We used the term "four-note chords with extensions" as the descriptor for the medium complexity chords used in our study. The stimuli consisted of various four-note D major chords with an additional fifth note, or an 'extension.' In this case, the term 'extension' comes from jazz theory (Gross. 2015) and refers to a note that goes above the octave (e.g., a 9th or a 13th). So, the chord illustrated in figure 1 would be an example of a four-note chord with an extension; in particular, it would be a ninth chord.

We have adjusted the figure caption to coincide better with how these stimuli were used in previous work (Matthews et al., 2019; Witek et al., 2023).

References:

Gross, A. (2015). Jazz Theory: From Basic to Advanced Study.

Matthews, T. E., Witek, M. A., Heggli, O. A., Penhune, V. B., & Vuust, P. (2019). The sensation of groove is affected by the interaction of rhythmic and harmonic complexity. *PLoS One*, *14*(1), e0204539.

Witek, M. A., Matthews, T., Bodak, R., Blausz, M. W., Penhune, V., & Vuust, P. (2023). Musicians and non-musicians show different preference profiles for single chords of varying harmonic complexity. *Plos One*, *18*(2), e0281057.

For comprehensiveness, I would like to see all--or at least a strong sampling--of musical examples included in the Appendix. Including links to sound samples would be even better, so the reader can test the various stimuli themselves.

We have included links in the supplementary materials so that individuals can access all of the sound samples used in our study.

Finally, I have a question about "the dampening of overall pleasure" discussed in line 486. For the control group, "pleasure" in these samples is presumably due to several causes, whereas--as you explain--for the anhedonic group, it only comes from the urge to move. Is it possible that this explains the "dampening of overall pleasure"? I.e. Both the control group and anhedonics get the same amount of pleasure from the urge to move; but the control group adds additional sources of pleasure? Or am I misunderstanding in some way?

We agree with the reviewer's understanding of our interpretation of these findings.

Reviewer #3: The author has at least made changes to the manuscript in detail and thoroughly, the use of language also meets writing standards. The author has fulfilled the completeness of supporting data

We thank reviewer #3 for their comments.
